# Anticonvulsant Effects of Citronellal, Daidzin, and Phytol, Possibly Through GABA_A_ Receptor and Voltage‐Gated Sodium Channel Interaction Pathways: In Vivo and In Silico Studies

**DOI:** 10.1002/brb3.71415

**Published:** 2026-04-16

**Authors:** Emon Mia, Md. Anisur Rahman, Md. Nasimul Haque Shipon, Moushumi Afroza Mou, Proma Mandal, Imam Hossen Rakib, Md. Abu Sayeed, Muhammad Torequl Islam, Md. Sakib Al Hasan, Mushtaq Ahmad Ansari

**Affiliations:** ^1^ Department of Pharmacy Gopalganj Science and Technology University Gopalganj Bangladesh; ^2^ Bioinformatics and Drug Innovation Laboratory BioLuster Research Center Ltd. Gopalganj Bangladesh; ^3^ Department of Pharmacy Islamic University Kushtia Bangladesh; ^4^ Department of Biological Science St. John's University, Queens New York New York USA; ^5^ Department of Pharmaceutical Sciences College of Pharmacy and Health Sciences St. John's University New York New York USA; ^6^ Pharmacy Discipline Khulna University Khulna Bangladesh; ^7^ Department of Pharmacology and Toxicology College of Pharmacy King Saud University Riyadh Saudi Arabia; ^8^ Alor Dishari Research Organization Dhaka Bangladesh; ^9^ BUBT Research Graduate School Bangladesh University of Business and Technology Dhaka Bangladesh; ^10^ Department of Pharmacy Mawlana Bhashani Science and Technology University, Santosh Tangail Bangladesh

**Keywords:** anticonvulsant effect, GABA receptor, natural products, sodium channel

## Abstract

**Background:**

Natural compounds, such as citronellal (CTL), daidzin (DZN), and phytol (PHY), have demonstrated potential anticonvulsant properties, but their combined effects remain unexplored. This study aimed to evaluate the anticonvulsant efficacy of CTL, DZN, and PHY, individually and in combination, using in vivo and in silico approaches.

**Methods:**

In the in vivo study, pentylenetetrazole (PTZ)‐induced seizures in chicks were used to assess seizure latency, frequency, and duration. CTL (250 mg/kg), DZN (20 mg/kg), and PHY (75 mg/kg) were administered oral (p.o.), alone and in combination. Carbamazepine (CAR, 80 mg/kg) and diazepam (DZP, 5 mg/kg) served as standard controls. In silico molecular docking was performed against γ‐aminobutyric acid A (GABA_A_) and voltage‐gated sodium channel (VGSC), whereas pharmacokinetics and toxicity predictions were evaluated.

**Results:**

Results showed that CTL, DZN, and PHY significantly increased seizure latency, reduced seizure frequency, and shortened seizure duration. The combination of CTL + DZN + PHY enhanced these effects by increasing latency and reducing seizure frequency and duration. When combined with DZP, the combination provided the highest protection (83.33%) and significantly reduced seizure duration. Docking studies revealed strong binding affinities (BAs) to GABA_A_ (CTL: −7.0 kcal/mol, DZN: −8.4 kcal/mol, PHY: −5.4 kcal/mol) and VGSC (DZN: −8.9 kcal/mol). Pharmacokinetic analysis confirmed good absorption and blood–brain barrier (BBB) permeability, whereas toxicity profiling indicated a high safety margin.

**Conclusion:**

These findings suggest that CTL, DZN, and PHY exert significant anticonvulsant effects via GABAergic and sodium channel modulation, warranting further clinical investigation.

## Graphical abstract

Citronellal, daidzin, and phytol exert significant anticonvulsant effects in a chick seizure model by enhancing GABAergic transmission and blocking voltage‐gated sodium channels, with combination therapies showing enhanced efficacy and a favorable safety profile.

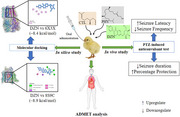

## 1 Introduction

Epilepsy is characterized by abnormal neurotransmitter activity in the central nervous system (CNS), causing neuronal instability and resulting in seizures and related symptoms (Akram et al. 2023). The disease can develop and manifest in individuals of all ages, from newborns to the elderly; however, it is most commonly diagnosed in children under the age of 10 and in adults over 85 years old (Beghi 2020). Globally, epilepsy affects approximately 50 million people, with 5 million new cases annually, often leading to reduced quality of life and psychological challenges like anxiety and depression (Alimoradi et al. 2023; Pontes Silva and Gama Marques 2023). It can arise from hereditary factors or brain injuries such as head trauma, stroke, vascular abnormalities, congenital brain deformities, or CNS infections (Stafstrom and Carmant 2015). It is a heterogeneous condition, with types differing in etiology, symptoms, and treatment response (Scheffer et al. 2017). Pharmacotherapy is the main treatment for epilepsy, using antiseizure medications (ASMs) that modulate ion channels (e.g., sodium, calcium, potassium), enhance GABA neurotransmission, inhibit excessive glutamate activity, or regulate neurotransmitter release, improving seizure control and quality of life (Sills and Rogawski 2020; Łukasiuk and Lasoń 2023).

Although most epilepsy patients achieve seizure control with antiepileptic drugs (AEDs), over 30% experience medically refractory epilepsy (Kwan and Brodie 2000; Verrotti et al. 2020). Furthermore, approximately 30%–40% of all epilepsy patients continue to experience various side effects and seizure resistance with the current AEDs (Sørensen and Kokaia 2013). AEDs work primarily by promoting inhibitory processes above excitatory ones, which halt or prevent seizure activity from spreading (Rogawski et al. 2016). Currently, conventional drugs commonly used for treating epilepsy include carbamazepine (CAR), valproate sodium, phenobarbital (PB) sodium, phenytoin sodium, and prelampone (Fu and Qu 2019). These drugs regulate excitatory and inhibitory brain discharges by adjusting ion concentrations. Phenytoin sodium is highly effective but can cause anemia and cerebellar ataxia. CAR may lead to rashes, neurotoxicity, and blood or respiratory issues (He et al. 2021). Newer AEDs, like topiramate, lamotrigine, levetiracetam, and gabapentin, offer broader effects with fewer side effects but may not fully address refractory epilepsy, whereas natural medicines maintain the biological functions of their components (Guo et al. 2015; Singh et al. 2020).

Natural products are widely explored in animal health due to their diverse bioactivity; however, their safety should not be assumed, as they may cause toxicity, organ damage, or drug–herb interactions in susceptible individuals. Although they may not always be as potent as conventional drugs, they can support therapy and may reduce side effects when used as adjuncts (Yuan et al. 2019). The current study highlights the antiepileptic effects of natural drug components, including flavonoids, alkaloids, glycosides, coumarins, and terpenoids, with flavonoids, alkaloids, and terpenoids showing significant activity (He et al. 2021).

Citronellal (CTL), a monoterpene produced through the secondary metabolism of plants such as *Corymbia citriodora*, *Cymbopogon nardus*, and *Cymbopogon winterianus*, is typically obtained as a non‐racemic mixture of its R and S enantiomers through steam distillation or solvent extraction (Lenardão et al. 2007). CTL has shown a broad range of pharmacological effects, including CNS depressant and anticonvulsant properties (Quintans‐Júnior et al. 2008), hypotensive and vasorelaxant effects (de Menezes et al. 2010), lipid‐lowering (Paul et al. 2022), cardioprotective (Liu et al. 2022), hepatoprotective (Malik et al. 2024), anti‐inflammatory (Melo et al. 2011), antidiabetic (Qiu et al. 2021), antinociceptive (Melo et al. 2010), antibacterial (Guliani et al. 2018), antifungal (Ana et al. 2017), anthelmintic (Araújo‐Filho et al. 2019), sedative (Jäger et al. 1992), antioxidant (Yousefi et al. 2019), antidepressant (Victoria et al. 2014), anesthetic (Hoseini et al. 2022), and anticancer properties (Ho et al. 2020). Recent studies have also highlighted its CNS depressant and antinociceptive effects (Melo et al. 2010; Quintans‐Júnior et al. 2010). CTL exhibits BA for the GABA_A_ receptor and forms several linkages for the VGSC receptor, contributing to its anticonvulsant effects (Chowdhury et al. 2024).

Daidzin (DZN), a phytoestrogen isoflavone derived from soy, shares structural similarities with estrogen (Sirotkin et al. 2021). DZN has demonstrated diverse biological activities, including anticancer (Yao et al. 2021), cardioprotective (Vadivelan et al. 2022), antidiabetic (Singla et al. 2024), anti‐osteoporotic (Martiniakova et al. 2020), skin protective (Ubaid et al. 2023), hepatoprotective (Kim et al. 2009), and nephroprotective (Wu et al. 2018) effects. Additionally, it has neurological benefits such as memory enhancement (Kim et al. 2010) and antiepileptic effects (Kazmi et al. 2020). DZN also interacts with the GABA_A_ receptor, demonstrating significant BA and sedative effects in Swiss mice (Islam, Al Hasan, Chowdhury, et al. 2024).

Phytol (PHY), a diterpene found in chlorophyll and vitamins K and E and used in fragrance compounds, shows a variety of pharmacological activities. These include antioxidant (Santos et al. 2013), anti‐cholinesterase (Sathya et al. 2018), anti‐amyloidogenic (Neha et al. 2024), anti‐quorum sensing (Srinivasan et al. 2017), anticancer (de Alencar et al. 2019), and anti‐inflammatory (Carvalho et al. 2020) effects. PHY has also been implicated in regulating neuronal activity, affecting neurotransmitter systems, and adjusting the synthesis and/or release of inhibitory neurotransmitters involved in the seizure process (Costa et al. 2012). In addition, PHY can have a sedative‐like effect on TS‐induced sleeping rats by interacting with the GABA_A_ receptor (Islam et al. 2015; IFRA 2004).

Combination therapy is the use of two or more treatments together to enhance effectiveness, reduce resistance, or target multiple aspects of a disease. It is commonly used in cancer, infectious diseases, chronic conditions, and mental health (Makhoba et al. 2020; Sauter 2020). CTL, DZN, and PHY are known to exhibit anticonvulsant effects individually. However, the potential synergistic effects of their combination have not yet been investigated.

This study aims to explore the anti‐convulsant effects of the compounds CTL, DZN, and PHY, combining molecular docking, pharmacokinetics, and toxicity profiles to assess their potential efficacy and safety.

## 2 Materials and Methods

### 2.1 In Vivo Study

#### 2.1.1 Chemicals and Reagents

DZN, with a purity of 98% HPLC (CAS: 552‐66‐9), was obtained from Chengdu Alfa Biotechnology Co. Ltd. (China). Diazepam (DZP) (Sedil: 5 mg tablet) and CAR (Anleptic: 100 mg/5 mL oral suspension) were generously provided by Square Pharmaceuticals Ltd. (Bangladesh). CTL, with a purity of ≥95% GC (CAS No. 106‐23‐0), and PHY (CAS: 7541‐49‐3, purity: 97% HPLC) were sourced from Sigma‐Aldrich (Germany). Tween 80 and NaCl, required for this research, were purchased from Merck (India).

#### 2.1.2 Experimental Animals

Young broiler chicks (*Gallus gallus domesticus*) of both sexes, weighing between 38–40 g and 2 days old, were purchased from a local market in Khulna, Bangladesh. Young chicks were used as an established and sensitive behavioral model for screening CNS‐active compounds, allowing rapid assessment under controlled conditions (Islam, Ahammed, et al. 2025). The chicks were acclimatized for 2 days in the pharmacology lab at Gopalganj Science and Technology University (GSTU) before the study began. During this period, they had unrestricted access to standard food and water. The room temperature was maintained at 27°C ± 2°C with a 12‐h light/dark cycle under controlled lighting. The study was conducted after a 12‐h fasting period, with the chicks only having access to water. Experiments were carried out between 9:00 a.m. and 3:00 p.m. This study was approved by the Animal Ethics Committee of Khulna University (KUAEC‐2024‐05‐09). The procedures adhered to ARRIVE guidelines (https://arriveguidelines.org) for reporting and conducting animal research.

#### 2.1.3 Dose Selection and Study Design

The test doses used in this study for CTL (250 mg/kg), DZN (20 mg/kg), and PHY (75 mg/kg) were selected on the basis of previous research by Melo et al. (2010), Kazmi et al. (2020), and Costa et al. (2012), respectively. The doses used in chicks were selected on the basis of previously published pharmacological studies and were adapted for avian body weight and physiology using standard interspecies dose‐conversion principles. Animals were randomly assigned and observations were recorded in a blinded manner. CAR (80 mg/kg) and DZP (5 mg/kg) were administered as per literature (Chowdhury et al. 2024). The control group received normal saline containing 0.5% Tween 80 at a dose of 10 mL/kg. All treatments were given p.o. Prior to treatment, all animals underwent overnight fasting. Table 1 outlines the animal grouping and treatment protocol.

**TABLE 1:** Treatment groups with their details at 10 mL/kg volume via oral route.

Treatment groups	Description	Administration design
**Individual groups**		
Control	Vehicle: distilled water containing 0.9% NaCl and 0.5% Tween 80	At a time
CAR80	Carbamazepine (voltage‐gated sodium channel reference drug) 80 mg/kg	At a time
DZP5	Diazepam (GABAergic reference drug) 5 mg/kg	At a time
CTL250	Citronellal (test sample 1) 250 mg/kg	At a time
DZN20	Daidzin (test sample 2) 20 mg/kg	At a time
PHY75	Phytol (test sample 3) 75 mg/kg	At a time
**Combination groups**		
CTL250 + DZN20 + PHY75	Citronellal 250 mg/kg + Daidzin 20 mg/kg + Phytol 75 mg/kg	One followed by another
CTL250 + DZN20 + PHY75 + CAR80	Citronellal 250 mg/kg + Daidzin 20 mg/kg + Phytol 75 mg/kg + Carbamazepine 80 mg/kg	One followed by another
CTL250 + DZN20 + PHY75 + DZP5	Citronellal 250 mg/kg + Daidzin 20 mg/kg + Phytol 75 mg/kg + Diazepam 5 mg/kg	One followed by another

*Note*: Control: Vehicle (distilled water containing 0.9% NaCl and 0.5% Tween 80). p.o.: oral (*n* = 6).

Abbreviations: CAR, Carbamazepine; CTL, Citronellal; DZN, Daidzin; DZP, Diazepam; PHY, Phytol.

#### 2.1.4 Pentylenetetrazole (PTZ)‐Induced Convulsion Test

This study followed the method outlined by Herrera‐Calderon et al. (2018) with minor modifications. Treatments were administered in the same manner, and after a 30‐min period, PTZ was injected intraperitoneally (i.p.) at a dose of 80 mg/kg. Animals were then observed continuously for 10 min after PTZ administration. The onset of convulsion (OC) was recorded as the time to the first convulsion, seizure frequency was recorded as the total number of seizure episodes within the 10‐min observation period, and the duration of convulsion (DC) was noted as the mean duration of individual seizure episodes, along with the number of fatalities. The percentage protection (%) was calculated by using the following formula:

$$\%\mathrm{Protection}=\left[\left\{\left(\mathrm{Anima}l_{\mathrm{Used}}-\mathrm{Anima}l_{\mathrm{Death}}\right)÷\mathrm{Anima}l_{\mathrm{Used}}\right\}\times 100\right]$$

#### 2.1.5 Statistical Analysis

Values are expressed as the mean ± standard error of the mean (SEM). One‐way ANOVA (analysis of variance) followed by a Tukey post hoc multiple comparison test at 95% confidence intervals using GraphPad Prism software (version: 9.5, San Diego, USA). Data were considered significant when *p* < 0.05.

### 2.2 In Silico Studies

#### 2.2.1 Selection and Preparation of Macromolecules

For molecular docking and protein–ligand visualization, we focused on the GABA_A_ and VGSC receptors, which are implicated in seizures and convulsions, as reported in the literature (Chowdhury et al. 2024). The GABAA receptor (PDB ID: 6X3X) and the VGSC receptor (PDB ID: 8S9C) were obtained from the RCSB Protein Data Bank (https://www.rcsb.org/). The receptors were optimized using PyMol (version 2.4.1) to minimize docking interferences by removing unnecessary protein chains, lipids, water molecules, and heteroatoms (Islam, Al Hasan, Ferdous, et al. 2025c). The structures were saved in PDBQT format for docking, and energy minimization was performed using SwissPDB Viewer software.

#### 2.2.2 Preparation of Ligands

**FIGURE 1:**
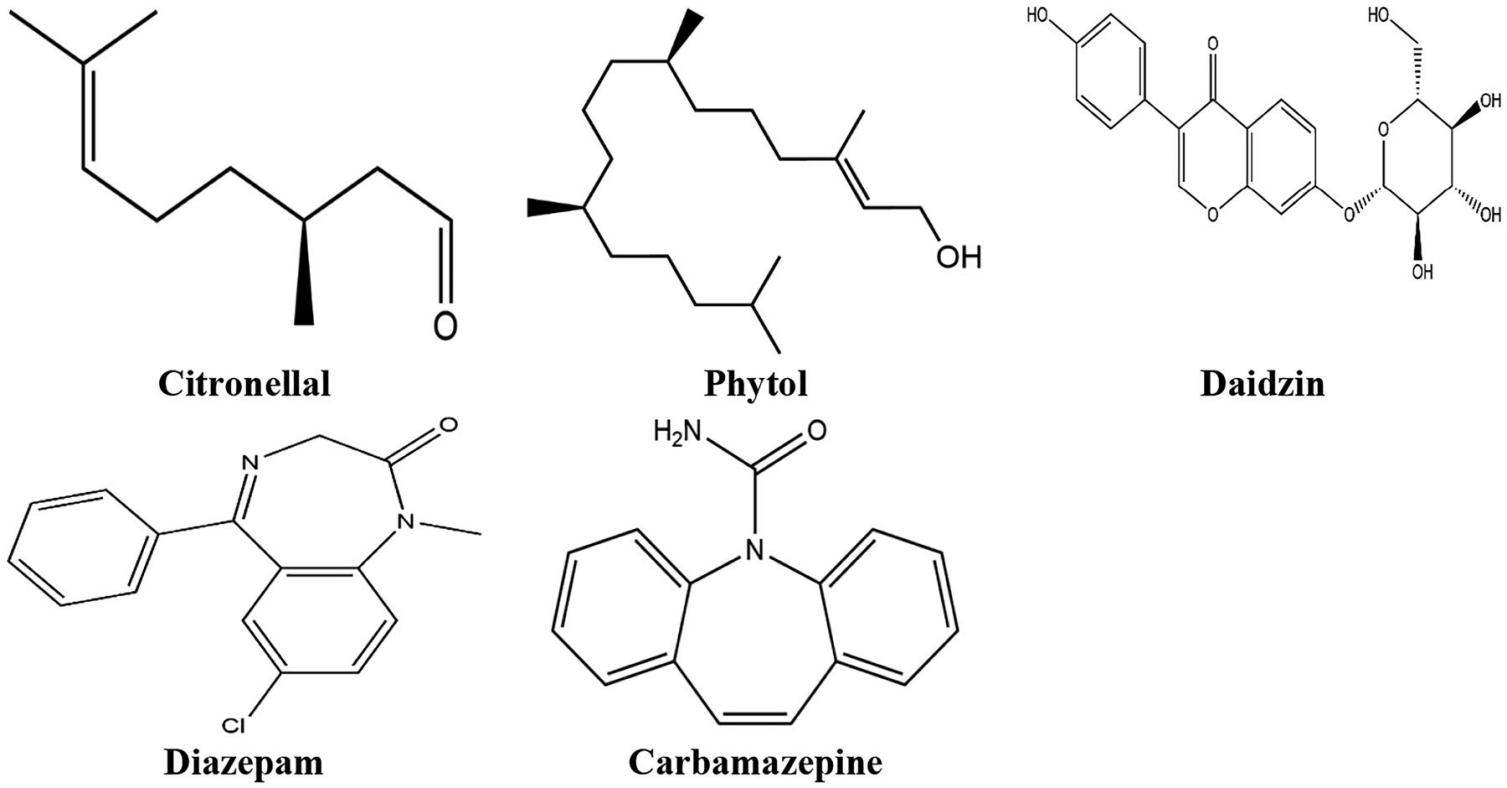
The 2D structure of the test compounds Citronellal, Phytol, Daidzin and standards Carbamazepine, and Diazepam.

The 3D conformers of CTL (Compound ID: 7794), DZN (Compound ID: 107971), CAR (Compound ID: 2554), DZP (Compound ID: 3016), and PHY (Compound ID: 5280435) were retrieved from the PubChem database (https://pubchem.ncbi.nlm.nih.gov/) and saved in SDF format. The ligands were then minimized and saved in SDF format using Chem3D 16.0. The 2D structures of the compounds are shown in Figure 1.

#### 2.2.3 Molecular Docking and Visualization

Molecular docking was performed using the PyRx software tool to assess the active binding capability of the drugs against receptor sites of activity (Bhuia et al. 2023). The protein–ligand complex was saved in PDB format, and the BAs were recorded in a CSV file. Protein–ligand interactions within the active site of the macromolecule were identified and analyzed using PyMol (v2.4.1) and Discovery Studio Visualizer (v21.1.020298) (Hasan et al. 2024). The amino acid residues involved in each ligand–receptor interaction, along with bond types, hydrogen bonds (HBs), HB lengths, and other bond types, were documented.

## 3 Results

### 3.1 PTZ‐Induced Anticonvulsant Test

#### 3.1.1 Seizure Latency

In our studies, the control group had a latency of 21.33 ± 0.99 s. CAR80, DZP5, and DZN20 treatments moderately increased latency, reaching 25.50 ± 0.99, 27.50 ± 2.92, and 32.50 ± 1.38 s, respectively. These increases were significant compared to the control (*p* < 0.05), indicating a delayed onset of seizures. However, CTL250 and PHY75 markedly improved latency times to 129.17 ± 5.39 and 126.33 ± 8.35 s, respectively, showing significant differences (*p* < 0.05) compared to control, CAR80, and DZP5 groups. The combination of CTL250 + DZN20 + PHY75 significantly enhanced latency to 102.83 ± 13.39 s, which was lower than the individual CTL250 or PHY75 treatments but still significant (*p* < 0.05) compared to control, CAR80, and DZP5. The co‐treatment with CTL250 + DZN20 + PHY75 + CAR80 further extended latency to 113.50 ± 4.39 s, showing an improvement compared to the three‐drug combination. Surprisingly, the co‐treatment with CTL250 + DZN20 + PHY75 + DZP5 resulted in a latency reduction (8.00 ± 2.19 s), significantly lower than individual or other co‐treatment results.

#### 3.1.2 Seizure Frequency

The seizure frequency in the control group was high, at 70.17 ± 2.77. CAR80 reduced this frequency to 39.00 ± 2.93, whereas DZP5 further reduced it to 18.50 ± 1.48. DZN20 showed a similar reduction to DZP5 (18.33 ± 1.20). CTL250 and PHY75 had the most pronounced effect among individual treatments, reducing seizure frequency to 9.33 ± 0.88 and 5.17 ± 0.79, respectively, with statistically significant differences from control, CAR80, and DZP5 groups. The three‐compound co‐treatment (CTL250 + DZN20 + PHY75) reduced seizure frequency to 14.17 ± 1.14 compared to control, showing a less reduction than individual treatments with CTL250 or PHY75. When CAR80 was added to the three‐compound combination, the seizure frequency decreased further to 6.83 ± 1.22, which was comparable to PHY75 alone. The combination of CTL250 + DZN20 + PHY75 + DZP5 also demonstrated the significant reduction in seizure frequency, reaching 7.17 ± 1.58, indicating a potent combined effect. This combination resulted in a statistically significant improvement compared to CAR80, DZP5, and other co‐treatment groups (Table 2).

**TABLE 2:** Effect of test samples and/or controls on pentylenetetrazole‐induced behaviors after oral administration at 10 mL/kg.

Treatment groups	Latency (s)	Frequency	Duration (s)
**Individual treatment groups**			
Control	21.33 ± 0.99	70.17 ± 2.77	367.33 ± 13.06
CAR80	25.50 ± 0.99*	39.00 ± 2.93*	256.00 ± 8.83*^d^
DZP5	27.50 ± 2.92*^a^	18.50 ± 1.48*^a^	245.33 ± 13.65*^ad^
CTL250	129.17 ± 5.39*^abde^	9.33 ± 0.88*^abd^	56.50 ± 8.00*^abde^
DZN20	32.50 ± 1.38*^ab^	18.33 ± 1.20*^a^	267.33 ± 13.81*
PHY75	126.33 ± 8.35*^abd^	5.17 ± 0.79*^abcd^	119.67 ± 5.16*^abd^
**Co‐treatment groups**			
CTL250 + DZN20 + PHY75	102.83 ± 13.39*^abd^	14.17 ± 1.14*^abd^	182.67 ± 7.78*^abd^
CTL250 + DZN20 + PHY75 + CAR80	113.50 ± 4.39*^abd^	6.83 ± 1.49*^abcd^	76.33 ± 7.82*^abde^
CTL250 + DZN20 + PHY75 + DZP5	8.00 ± 2.19	7.17 ± 1.58*^abcd^	15.50 ± 2.43*^abcde^

*Note*: Values are mean ± SEM (*n* = 6); one‐way ANOVA followed by a Tukey multiple comparison test; *p* < 0.05 compared to the *Control, ^a^CAR80, ^b^DZP5, ^c^CTL250, ^d^DZN20, and ^e^PHY75 groups; Control: Vehicle (distilled water containing 0.9% NaCl and 0.5% Tween 80).

Abbreviations: CAR, Carbamazepine; CTL, Citronellal; DZN, Daidzin; DZP, Diazepam; PHY, Phytol.

#### 3.1.3 Seizure Duration

Control group animals had a seizure duration of 367.33 ± 13.06 s. CAR80, DZP5, and DZN20 treatments all significantly decreased seizure duration to 256.00 ± 8.83, 245.33 ± 13.65, and 267.33 ± 13.81 s, respectively, compared to the control group (*p *< 0.05). CTL250 and PHY75 treatments had the greatest impact in reducing seizure duration, showing values of 56.50 ± 8.83 and 119.67 ± 5.16 s, respectively, against control, CAR80, DZP5, and DZN20 (*p* < 0.05). The three‐compound combination (CTL250 + DZN20 + PHY75) resulted in a seizure duration of 182.67 ± 7.78 s, which was longer than individual CTL250 or PHY75 treatment. The addition of CAR80 to the three‐compound combination further shortened the duration to 76.33 ± 7.83 s, showing a significant reduction from the three‐drug combination alone (*p* < 0.05). Notably, the co‐treatment with CTL250 + DZN20 + PHY75 + DZP5 yielded the shortest duration (15.50 ± 2.43 s), indicating that adding DZP5 to the combination may enhance the anticonvulsant effects (Table 2).

#### 3.1.4 Percentage Protection

The control group displayed no protection (0.00%), whereas individual treatments such as DZP5 and PHY75 offered the highest protection at 83.33%, which was statistically significant compared to the control (*p* < 0.05). CAR80, DZN20, and CTL250 showed moderate protection levels at 67.67%. These values indicate partial efficacy but not as strong as DZP5 or PHY75. Co‐treatment groups provided similar protection to DZP5 and PHY75 alone. Notably, the combination of CTL250 + DZN20 + PHY75 + DZP5 maintained an 83.33% protection level, indicating no significant difference compared to the individual use of DZP5 or PHY75. Therefore, the summarized data are presented in Table 3.

**TABLE 3:** The percentage protection of chicks in different treatments.

Treatment groups	Percentage protection
**Individual treatment groups**	
Control	0.00
CAR80	67.67
DZP5	83.33
CTL250	67.67
DZN20	67.67
PHY75	83.33
**Co‐treatment groups**	
CTL250 + DZN20 + PHY75	67.67
CTL250 + DZN20 + PHY75 + CAR80	67.67
CTL250 + DZN20 + PHY75 + DZP5	83.33

*Note*: Control: vehicle (distilled water containing 0.9% NaCl and 0.5% Tween 80).

Abbreviations: CAR, Carbamazepine; CTL, Citronellal; DZN, Daidzin; DZP, Diazepam; PHY, Phytol.

### 3.2 In Silico Findings

#### 3.2.1 Molecular Docking of CTL, PHY, and DZN With 6X3X Receptor

For the 6X3X receptor, the BAs of CTL, PHY, and DZN were −7.0, −5.4, and −8.4 kcal/mol, respectively, whereas DZP had a BA of −7.1 kcal/mol. The number of HBs for CTL was one (with THR D: 207 at 1.99 Å), whereas DZN, PHY, and DZP did not form HBs. CTL exhibited interactions with residues such as TYR D: 210, PHE D: 100, TYR D: 160, TYR E: 58, and PHE E: 77. DZN interacted with residues like VAL B: 257, ALA A: 252, ALA C: 252, VAL D: 257, and LEU C: 259. PHY demonstrated interactions with residues such as TYR A: 304, LEU A: 301, LEU A: 297, VAL B: 243, ALA A: 300, TYR A: 304, and TRP B: 246. DZP showed interactions with residues THR E: 146 and LYS E: 118.

#### 3.2.2 Molecular Docking of CTL, PHY, and DZN With 8S9C Receptor

**FIGURE 2:**
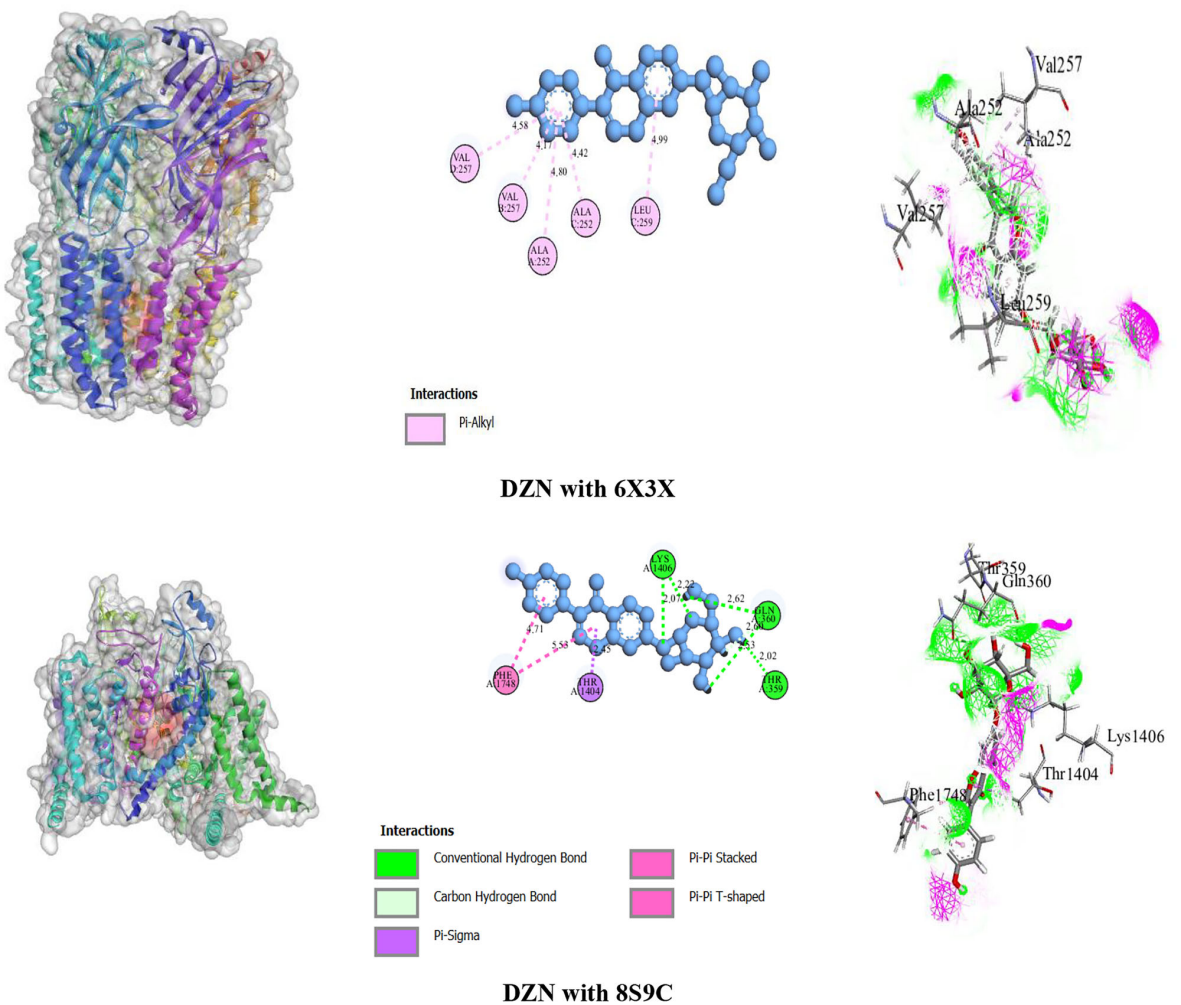
The 2D and 3D binding interactions of the Daidzin, with GABA_A_ (6X3X) and VGSC (8S9C) receptors.

For the 8S9C receptor, the BAs for CTL, PHY, DZN, and CAR were −5.5, −6.7, −8.9, and −6.8 kcal/mol, respectively. DZN had the highest BA, followed by CAR, PHY, and CTL. The number of HBs formed by DZN was 4, involving GLN A: 360 (2.61, 2.53 Å), THR A: 359 (2.01 Å), and LYS A: 1406 (2.07, 2.21 Å). In contrast, CTL, PHY, and CAR did not form HBs. CTL exhibited interactions with residues such as PHE A: 1446, ILE A: 1442, LEU A: 924, VAL A: 959, LEU A: 962, LEU A: 873, and PHE A: 1447. DZN interacted with residues, including GLN A: 360, THR A: 359, LYS A: 1406, THR A: 1404, and PHE A: 1748. PHY demonstrated interactions with residues such as PHE A: 843, PHE A: 1443, TRP A: 808, ILE A: 856, ILE A: 859, LEU A: 840, ILE A: 1334, PHE A: 811, and PHE A: 1335. CAR interacted with residues, including TRP A: 1332, LEU A: 1400, LEU A: 1449, LEU A: 1325, CYS A: 1328, LEU A: 1329, TYR A: 1396, PHE A: 1452, and PHE A: 1748. However, details of the BA, number of HB and AA residues related to HB, and other types of bonds of ligands with selected receptors are presented in Table 4. The binding pockets’ 2D and 3D structures, including the interacting AA residues and bond types of ligands with GABA_A_ (6X3X) and VGSC (8S9C) receptors, are depicted in Figure 2.

**TABLE 4:** Presents the number, length, and type of hydrogen bonds, along with the amino acid residues involved, between the selected ligands Citronellal, Phytol, Daidzin, Carbamazepine, and Diazepam with GABA_A_ (6X3X) and VGSC (8S9C) receptors.

Receptors (PDB ID)	Ligands	Binding affinity (kcal/mol)	No. of HB	Amino acid residues	
				Hydrogen bond with length (Å)	Other bonds
6X3X	CTL	−7.0	1	THR D: 207 (1.99)	TYR D: 210, PHE D: 100, TYR D: 160, TYR E: 58, PHE E: 77
	DZN	−8.4	—	—	VAL B: 257, ALA A: 252, ALA C: 252, VAL D: 257, LEU C: 259
	PHY	−5.4	—	—	TYR A: 304, LEU A: 301, LEU A: 297, VAL B: 243, ALA A: 300, TYR A: 304, TRP B: 246
	DZP	−7.1	—	—	THR E: 146, LYS E: 118
8S9C	CTL	−5.5	—	—	PHE A: 1446, ILE A: 1442, LEU A: 924, VAL A: 959, LEU A: 962, LEU A: 873, PHE A: 1446, PHE A: 1447
	DZN	−8.9	4	GLN A: 360 (2.61, 2.53) THR A: 359 (2.01) LYS A: 1406 (2.07, 2.21)	THR A: 1404, PHE A: 1748,
	PHY	−6.7	—	—	PHE A: 843, PHE A: 1443, TRP A: 808, ILE A: 856, ILE A: 859, LEU A: 840, ILE A: 1334, TRP A: 808, PHE A: 811, PHE A: 133, PHE A: 1335
	CAR	−6.8	—	—	TRP A: 1332, LEU A: 1400, LEU A: 1449, LEU A: 1325, CYS A: 1328, LEU A: 1329, TRP A: 1332, TYR A: 1396, PHE A: 1452, PHE A: 1748

Abbreviations: CAR, Carbamazepine; CTL, Citronellal; DZN, Daidzin; DZP, Diazepam; PHY, Phytol.

#### 3.2.3 Pharmacokinetic and Drug‐Likeness Properties

In this study, with a bioavailability score of 0.55, all molecules conformed to Lipinski’s criteria.With molecular weights of 416.38 g/mol, DZN is substantially heavier than CTL and PHY, which have molecular weights of 154.25 and 296.53 g/mol, respectively. PHY has a moderate solubility, whereas CTL and DZN are soluble in water. All compounds adhere to Lipinski's Rule of Five.

**FIGURE 3:**
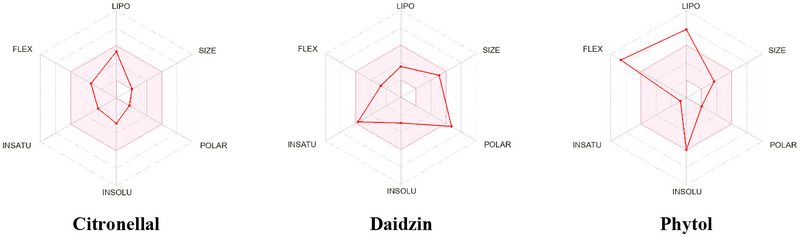
Bioavailability radar related to physicochemical properties of Citronellal, Daidzin, and Phytol (lipophilicity [LIPO]: −0.7 < XLOGP3 < +5.0, polarity [POLAR]: 20 Å^2^ < TPSA < 130 Å^2^, insolubility [INSOLU]: −6 < log *S* [ESOL] < 0, insaturation [INSATU] 0.25 < fraction Csp3 < 1, flexibility [FLEX]: 0 < Num, rotatable bonds <9).

CTL, DZN, and PHY showed varying pharmacokinetic properties. In terms of absorption, CTL and PHY exhibited high intestinal absorption (95.359% and 90.643%, respectively), whereas DZN had a lower absorption (59.319%). CTL demonstrated the best skin permeability (log *K*_p_: −1.386) compared to DZN (−2.736) and PHY (−2.631). For distribution, CTL and PHY showed positive blood–brain barrier (BBB) permeability (log BB: 0.648 and 0.793, respectively), whereas DZN had negative BBB permeability (−1.232). CTL and PHY also showed favorable volume of distribution (VDss) values (0.192 and 0.385 log L/kg, respectively), with DZN having a negative value (−0.166). None of the compounds were substrates or inhibitors of CYP2D6 or P‐glycoproteins, but only PHY was a CYP3A4 substrate. Regarding excretion, PHY had the highest total clearance (1.686 log mL/min/kg), followed by CTL (0.476), whereas DZN had the lowest (0.104). None of the compounds were renal OCT2 substrates. However, Table 5 and Figure 3 offer comprehensive details on pharmacokinetics, drug similarity, and the Lipinski Rule.

**TABLE 5:** Pharmacokinetics and drug‐*likeness* properties of Citronellal, Daidzin, and Phytol.

Properties	Factors	Citronellal	Daidzin	Phytol
**Physicochemical properties**	Formula	C_10_H_18_O	C_21_H_20_O_9_	C_20_H_40_O
	Molecular weight (g/mol)	154.25	416.38	296.53
	Number of heavy atoms	11	30	21
	Number of aromatic heavy atoms	0	16	0
	Number of H‐bond donors	0	5	1
	Number of H‐bond acceptors	1	9	1
	Molar refractivity	49.91	104.09	98.94
**Lipophilicity**	Log *P*_o/w_ (XLOGP3)	3.83	0.67	8.19
**Drug‐*likeness***	Lipinski	Yes; 0 violation	Yes; 0 violation	Yes; 1 violation: MLOGP >4.15
	Bioavailability score	0.55	0.55	0.55
**Water solubility**	Log *S* (ESOL)	−2.88	−2.97	−5.98
	Class	Soluble	Soluble	Moderately soluble
**Absorption**	Caco2 permeability (log *P*_app_ in 10^−6^ cm/s)	1.503	0.24	1.399
	Intestinal absorption (human) (%absorbed)	95.359	59.319	90.643
	Skin permeability (log *K*_p_ cm/h)	−1.386	−2.736	−2.631
	P‐glycoprotein I inhibitor	No	No	No
	P‐glycoprotein II inhibitor	No	No	No
**Distribution**	BBB permeability (log BB)	0.648	−1.232	0.793
	CNS permeability (log PS)	−2.049	−3.584	−1.527
	VDss (human) (log L/kg)	0.192	−0.166	0.385
**Metabolism**	CYP2D6 substrate	No	No	No
	CYP3A4 substrate	No	No	Yes
	CYP2D6 inhibitor	No	No	No
	CYP3A4 inhibitor	No	No	No
**Excretion**	Total clearance (log mL/min/kg)	0.476	0.104	1.686
	Renal OCT2 substrate	No	No	No

*Note*: H‐bond: hydrogen bond; CYP2D6 substrate: cytochrome P450 2D6 substrate; CYP3A4 substrate: cytochrome P450 3A4 substrate; CYP2D6 inhibitor: cytochrome P450 2D6 inhibition; CYP3A4 inhibitor: cytochrome P450 3A4 inhibition; renal OCT2 substrate: renal organic cation transporter 2 substrate.

Abbreviations: BBB, blood–brain barrier; CNS, central nervous system; VDss, volume of distribution.

#### 3.2.4 Toxicity Profile

The compounds CTL, DZN, and PHY have a toxicity class of 5 in the toxicological analysis. Furthermore, PHY (5000 mg/kg) showed higher LD_50_ than CTL (2420 mg/kg) and DZN (3100 mg/kg), suggesting that PHY has a superior safety profile. Because they were inactive in all of these areas, the toxicology investigation also showed that neither molecule had any adverse effects linked to hepatotoxicity, carcinogenicity, immunotoxicity, mutagenicity, or cytotoxicity. Table 6 lists several toxicity parameters for CTL, DZN, and PHY.

**TABLE 6:** Toxicity profiling of the Citronellal, Daidzin, and Phytol.

Toxicity	Parameters	Test sample		
		Citronellal	Daidzin	Phytol
	LD_50_	2420	3100	5000
	Toxicity class	5	5	5
	Hepatotoxicity	Inactive	Inactive	Inactive
	Carcinogenicity	Inactive	Inactive	Inactive
	Immunotoxicity	Inactive	Inactive	Inactive
	Mutagenicity	Inactive	Inactive	Inactive
	Cytotoxicity	Inactive	Inactive	Inactive

*Note*: LD_50_: median lethal dose.

## 4 Discussion

GABA imbalance is a major contributing factor to a number of neurological conditions, including epilepsy (Bayat et al. 2019). Levetiracetam, fosphenytoin, and valproate are commonly used second‐line anticonvulsants for managing convulsive status epilepticus (Kapur et al. 2019). DZP is effective for acute seizures (e.g., status epilepticus) but has short action and side effects like drowsiness, respiratory depression, tolerance, and dependence (Cornett et al. 2021). The anticonvulsant effect of (±) CTL likely involves enhancing GABAergic activity, which promotes neuronal inhibition, and modulating VGSCs, reducing neuronal excitability. These mechanisms help prevent seizure propagation (Chowdhury et al. 2024). Clobazam (Onfi), ezogabine (Potiga), perampanel (Fycompa), and eslicarbazepine (Aptiom) are the four new AEDs that the FDA has authorized for the treatment of epilepsy in the United States since 2010. Furthermore, two extended‐release versions have been approved: oxcarbazepine ER (Oxtellar) and topiramate ER (Qudexy XR and Trokendi XR) (Chong and Lerman 2016). *N*‐acetyl cysteine (NAC), when given as a pretreatment, reduces the severity of convulsions as measured by the Racine scale, which evaluates seizure intensity. This suggests NAC's properties may help mitigate seizure progression (Bilister Egilmez et al. 2023). CAR, a commonly used anticonvulsant medication for treating seizures, works through several mechanisms: it blocks sodium‐dependent channels, enhances the inhibitory activity of GABA, and diminishes the excitatory effects of glutamate receptors (Barzroodi Pour et al. 2021). CAR is a widely used AED, but its long‐term use poses risks such as reduced bone mineral density and altered lipid metabolism, alongside common side effects like nausea and dizziness (Koliqi et al. 2015).

PTZ is used to induce seizures in experimental models for studying epilepsy and testing anticonvulsant drugs (Bilister Egilmez et al. 2023). Felipe et al. (2019) revealed a study in which convulsions are induced using PTZ in order to assess the effectiveness of anticonvulsant medications (Felipe et al. 2019). Another study by Suleiman) also showed that convulsion can be induced by PTZ (Suleiman et al., 2025). Our study also revealed that PTZ can induce convulsion in young broiler chicks.

According to the in vivo study findings, in comparison to the control group, the animal's latency times for the different standard medications (CAR80, DZP5) were longer. More specifically, the latency times for DZP5 and CAR80 were 27.50 ± 2.92 and 25.50 ± 0.99 s, respectively. Notably, the CTL250, DZN20, and PHY75 treatment groups showed a latency period of 129.17 ± 5.39, 32.50 ± 1.38, and 126.33 ± 8.35 s, respectively, which was better than that of the standard drugs, DZP5 and CAR80. Our study observed that CTL250 and PHY75 displayed the most significantly prolonged latency period compared to both the standard drug and the control group.

**FIGURE 4:**
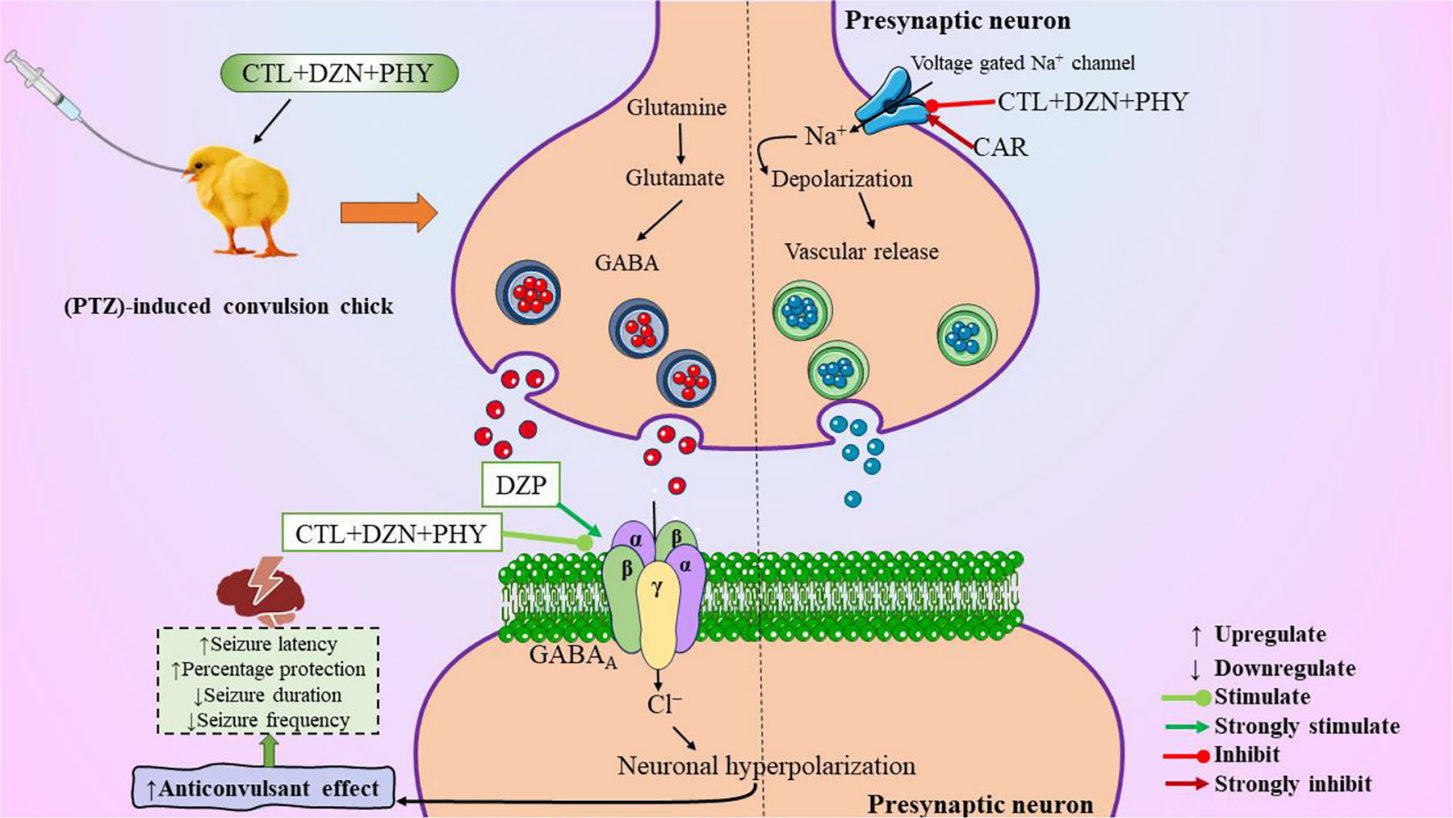
The possible mechanism of Citronellal, Daidzin, and Phytol on convulsion management. (This figure showed the potential anticonvulsant mechanism of Citronellal [CTL], Daidzin [DZN], and Phytol [PHY] in a PTZ‐induced convulsion model in chicks. These compounds appear to inhibit voltage‐gated sodium [Na^+^] channels in presynaptic neurons, reducing depolarization and neurotransmitter release. They also enhance GABAergic neurotransmission by increasing GABA release and stimulating GABA_A_ receptors, leading to chloride [Cl^−^] influx and neuronal hyperpolarization, which suppresses seizures. The overall anticonvulsant effects include increased seizure latency and protection, along with decreased seizure duration and frequency, suggesting a neuroprotective role in seizure management. The illustration was created using a template adapted and modified from Islam, Al Hasan, et al. [2025].) GABA, γ‐aminobutyric acid.

Combining several drugs may be more beneficial than using just one, according to some studies (Kwan and Brodie 2006). Compared to single medicine therapy, combination therapy has several advantages, such as a lower risk of drug resistance and notable synergistic effects that hasten recovery, which encourages researchers to explore the creation of novel medications (Bell 2013). Combination therapy demonstrated superior efficacy in reducing the emergence of drug resistance throughout prolonged treatment. If there is a high risk of repercussions, patients with chronic infectious diseases, such as cancer, diabetes, and tuberculosis, as well as those who have already developed medication resistance, may get combination therapy (Hassan et al. 2024). In our study, the combined treatment groups (CTL250 + DZN20 + PHY75) and (CTL250 + DZN20 + PHY75 + CAR80) showed significantly longer latency times than both the standard drug and the control group. CTL250 + DZN20 + PHY75 + DZP5 showed short latency times, but its short DC and number of frequencies revealed this combination as best for convulsion management. Potentiation of GABAergic neurotransmission is generally anticonvulsant; however, excessive enhancement of GABA‐mediated activity may paradoxically become pro‐ictogenic by disrupting network homeostasis and promoting disinhibition. This phenomenon could partly explain the shorter seizure latency observed with the combination of CTL250 + DZN20 + PHY75 + DZP5. However, a possible mechanism of this combination in convulsion is shown in Figure 4.

Previous study revealed that CTL exhibited dose‐dependent anticonvulsant effects in INH‐induced seizures, delaying seizure onset and reducing frequency and duration in mice, and it antagonized the effects of CAR and DZP in combination therapy (Chowdhury et al. 2024). Our study also supports this study by further studying it alone and in combination with DZP and CAR with the PTZ‐induced method. Another study revealed that DZN exerts antiepileptic and neuroprotective effects by modulating oxidative stress, BDNF/VEGF pathways, and apoptotic signaling in PTZ‐kindled mice (Kazmi et al. 2020). Our study revealed that DZN alone or in combination with DZP and CAR has been explored, unlike the above study, which did not assess combination effects, only highlighting DZN's antiepileptic potential via oxidative stress and apoptotic pathway modulation. The study by Costa et al. (2012) used DZP and PB as standard anticonvulsant drugs. PHY, when tested alone and in combination with these standards, enhanced seizure protection in the pilocarpine‐induced seizure model in mice (Costa et al. 2012). Our study explored the anticonvulsant effect in chicks using the PTZ‐induced model and demonstrated its effects both alone and in combination with convulsant.

Molecular docking is a structure‐based drug design technique that predicts how molecules interact and estimates the binding mechanism and affinity between ligands and receptors (Stanzione et al. 2021). Molecular docking has emerged as a crucial element in the drug discovery process (Stanzione et al. 2021). A BA higher than −6 kcal/mol suggests strong ligand–receptor interactions, indicating potential biological activity and drug‐like efficacy (Al Hasan et al. 2025). Stronger BA in drug development has been shown to increase potency, target selectivity, duration of action, and dose needs, all of which contribute to improved medication efficacy and safety (Tonge 2018). The molecular docking results indicate that DZN exhibited the highest BA among the tested compounds, surpassing both DZP and CAR in both receptors (6X3X: −8.4 kcal/mol, 8S9C: −8.9 kcal/mol). Compared to DZP (−7.1 kcal/mol) and CAR (−6.8 kcal/mol). CTL demonstrated moderate BA (6X3X: −7.0 kcal/mol, 8S9C: −5.5 kcal/mol), comparable to DZP in 6X3X but significantly lower in 8S9C. PHY exhibited the BA among the compounds (6X3X: −5.4 kcal/mol, 8S9C: −6.7 kcal/mol), indicating a lower BA potential relative to DZP and CAR.

HBs are typically regarded as key contributors to protein–ligand binding (Salentin et al. 2014). They play a vital role in stabilizing ligand–receptor complexes by providing strong, specific interactions. They enhance binding specificity and strengthen the overall interaction, ensuring precise molecular recognition (Du et al. 2023). DZN formed four HBs in the 8S9C receptor, contributing to its strong BA. In contrast, CTL formed a single HB in the 6X3X receptor, whereas PHY, DZP, and CAR did not form any HBs in either receptor.

The Lipinski Rule of Five predicts a compound's oral bioavailability based on molecular mass, lipophilicity, and hydrogen bonding properties (Nhlapho et al. 2024). The study of ADME (absorption, distribution, metabolism, and excretion) and therapeutic likeness evaluates a compound's behavior in the body and its potential as a safe, effective treatment, guiding dosing, delivery, and development strategies (Çevik et al. 2025). In our study, CTL, DZN, and PHY each exhibit distinct properties in relation to Lipinski's Rule of Five and other pharmacokinetic factors. CTL adheres to all Lipinski criteria with no violations, showing favorable drug‐likeness, good absorption (95.36%), and moderate bioavailability (0.55), along with favorable lipophilicity (log *P*_o/w_ = 3.83). DZN also complies with Lipinski's Rules but has moderate absorption (59.32%) and lower BBB permeability (−1.232), suggesting limited CNS activity despite its good solubility and bioavailability. PHY, violating Lipinski's Rule due to high lipophilicity (log *P*_o/w_ = 8.19), demonstrates high absorption (90.64%) but poor water solubility (−5.98), with a better potential for BBB penetration (log BB = 0.793), making it promising for CNS‐related applications despite its reduced drug‐likeness.

Toxicity is a crucial factor in drug discovery, as it helps determine the safety profile of potential drug candidates (Tran et al. 2023). In the case of CTL, DZN, and PHY, all three compounds belong to toxicity class five, indicating low toxicity. PHY, with the highest LD_50_ of 5000 mg/kg, demonstrates a superior safety profile compared to CTL (2420 mg/kg) and DZN (3100 mg/kg), with no adverse effects observed in tests for hepatotoxicity, carcinogenicity, immunotoxicity, mutagenicity, or cytotoxicity.

Our study investigated the anticonvulsant properties of CTL, DZN, and PHY using a PTZ‐induced seizure model in chicks, comparing their efficacy alone and in combination with standard drugs (DZP and CAR). Findings revealed that CTL250 and PHY75 significantly prolonged seizure latency, surpassing standard drugs, whereas combination therapies (CTL250 + DZN20 + PHY75) and (CTL250 + DZN20 + PHY75 + CAR80) exhibited even greater efficacy. Notably, the (CTL250 + DZN20 + PHY75 + DZP5) group showed shorter latency but effectively managed convulsions. Molecular docking supported these findings by highlighting strong ligand–target interactions, emphasizing HBs’ role in binding stability. Drug‐likeness and pharmacokinetics analysis reinforced the therapeutic potential of these compounds, aligning with Lipinski's Rule of Five and ADME studies. Toxicity profiles ensure all three compounds have low toxicity. Although our results suggest promising anticonvulsant effects, limitations include the use of a single seizure model, lack of in‐depth mechanistic studies, and the need for further validation in mammalian models to confirm clinical relevance. Although the chick model is useful for rapid CNS screening, rodent models may provide higher translational relevance, and future studies should validate these findings in mice or rats.

## 5 Conclusion

This study demonstrated that CTL, DZN, and PHY exhibit significant anticonvulsant effects individually and in combination as demonstrated in PTZ‐induced seizure models in chicks, which showed increased latency, reduced frequency, and shortened duration of seizures. The combination of CTL, DZN, and PHY enhanced anticonvulsant activity, and when combined with DZP, it provided the highest seizure protection (83.33%). Molecular docking studies revealed strong interactions with GABA_A_ receptors and VGSC, suggesting modulation of inhibitory neurotransmission and sodium channel blockade as key mechanisms. Pharmacokinetic analysis confirmed favorable absorption, BBB permeability, and metabolic stability, whereas toxicity profiling indicated a high safety margin. These findings highlight CTL, DZN, and PHY as promising anticonvulsant agents, warranting further research to optimize dosage, evaluate long‐term effects, and validate efficacy in higher animal models and clinical trials for potential use in epilepsy management.

## Author Contributions

**Emon Mia**: conceptualization, methodology, formal analysis, data curation, and writing – original draft preparation. **Md. Anisur Rahman**: methodology, investigation, and writing – original draft preparation. **Md. Nasimul Haque Shipon**: methodology, investigation, and writing – original draft preparation. **Moushumi Afroza Mou**: software, formal analysis, writing – review and editing. **Proma Mandal**: software, formal analysis, investigation, and visualization. **Imam Hossen Rakib**: software, formal analysis, investigation, and visualization. **Md. Abu Sayeed**: software, writing – review and editing. **Muhammad Torequl Islam**: conceptualization, resources, supervision, project administration, and writing – review and editing. **Md. Sakib Al Hasan**: conceptualization, resources, supervision, project administration, and writing – review and editing. **Mushtaq Ahmad Ansari**: formal analysis, resources, writing – review and editing, and funding acquisition. All authors have read and agreed to the published version of the manuscript.

## Funding

The authors were supported by the Ongoing Research Funding Program (ORF‐2026‐996), King Saud University, Riyadh, Saudi Arabia.

## Ethics Statement

This study was approved by the Animal Ethics Committee of Khulna University (KUAEC‐2024‐05‐09).

## Conflicts of Interest

The authors declare no conflicts of interest.

## Data Available Statement

The data that support the findings of this study are available from the corresponding author upon reasonable request.
